# Nanoscale optical interferometry with incoherent light

**DOI:** 10.1038/srep20836

**Published:** 2016-02-16

**Authors:** Dongfang Li, Jing Feng, Domenico Pacifici

**Affiliations:** 1 School of Engineering, Brown University, Providence, Rhode Island, 02912, United States

## Abstract

Optical interferometry has empowered an impressive variety of biosensing and medical imaging techniques. A widely held assumption is that devices based on optical interferometry require coherent light to generate a precise optical signature in response to an analyte. Here we disprove that assumption. By directly embedding light emitters into subwavelength cavities of plasmonic interferometers, we demonstrate coherent generation of surface plasmons even when light with extremely low degrees of spatial and temporal coherence is employed. This surprising finding enables novel sensor designs with cheaper and smaller light sources, and consequently increases accessibility to a variety of analytes, such as biomarkers in physiological fluids, or even airborne nanoparticles. Furthermore, these nanosensors can now be arranged along open detection surfaces, and in dense arrays, accelerating the rate of parallel target screening used in drug discovery, among other high volume and high sensitivity applications.

Optical interferometry based on surface plasmon polaritons (SPPs) has enabled a broad range of technical advantages^1^,^2^,^3^,^4^,^5^,^6^, including enhanced light transmission^7^,^8^, all-optical modulation^9^, light beaming^10^,^11^,^12^,^13^,^14^, and medical imaging^15^. More recently, control of SPPs interference at the nanoscale has led to biochemical sensors with unparalleled sensitivity and selectivity^16^,^17^,^18^,^19^. However, sensing architectures based on plasmonic interferometry typically require coherent external light sources that have to be carefully aligned and periodically calibrated, which poses challenges to reliable integrated devices. Here we develop an alternative design for a biochemical sensor that does not require a coherent external light source, precise alignment, or re-calibration. We have done so by embedding solid-state light emitters directly into the nanoaperture of plasmonic interferometers, where they serve as the localized light source. It is definitively shown that the optical response of this novel sensor is independent of coherence, spectral bandwidth, and incidence angle of the excitation source. This latter property is particularly advantageous when measuring chemical analytes in micro-droplets, where lensing effects can significantly vary the angle of incidence of the light beam, thus introducing artifacts in the sensor response.

Plasmonic interferometers consisting of a central hole flanked by three grooves (H-3G) with subwavelength width were fabricated on a 300 nm-thick silver film by focused ion beam milling and then coated with a thin (~40 nm) Cr^3+^:MgO light emitting layer^20^,^21^ (see Fig. 1a, Methods and Supplementary Fig. S1 for additional fabrication details). In contrast to other implementations of this structure (also known as bullseye^10^,^12^,^13^,^14^), here each excited emitter (i.e. Cr^3+^ ion in MgO) within the nanohole of each interferometer has a high probability to decay by generating SPP waves (with amplitude *E*_SPP_, dashed horizontal lines in Fig. 1a; also see Supplementary Fig. S2a)^22^,^23^. These SPPs propagate away from the nanohole and are then partially reflected back^24^,^25^ by the subwavelength grooves toward the nanohole, where they interfere with the directly emitted light (with amplitude *E*_0_, red solid arrow in Fig. 1a), thus modifying the fluorescence spectra in the far field. The fluorescence spectra transmitted through the nanohole can then be modulated by varying the interferometer arm length *R*_G_ or the refractive index of the dielectric material on the surface, thus providing for a novel sensing scheme. (See Supplementary Fig. S2 for more details.)

## Results

### Fluorescence modification induced by active plasmonic interferometry

**Figure 1:**
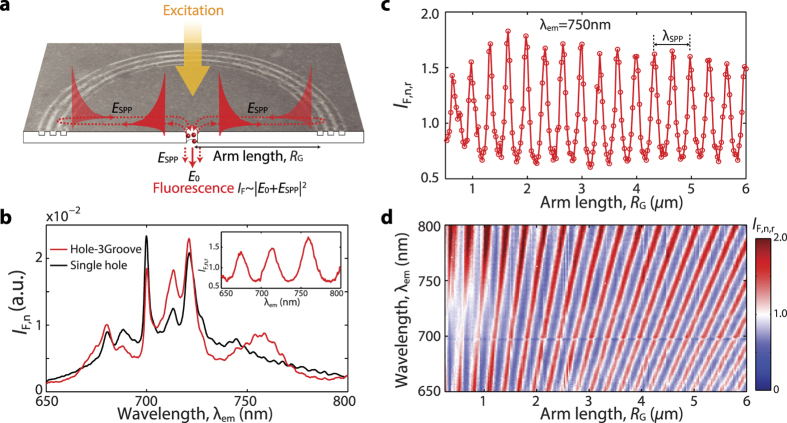
Active plasmonic interferometry with embedded light emitters. (**a**) Scanning electron micrograph (SEM) and schematic cross section of a circular hole-3groove (H-3G) plasmonic interferometer whose nanoaperture has been filled with Cr^3+^:MgO light emitters. Excited emitters in the nanohole (red dots) generate SPPs that undergo in-plane reflection by the nano-grooves. The fluorescence intensity detected in the far field is the result of the interference between the directly emitted field (*E*_0_, vertical solid arrow) and the transmitted field originating from diffractive scattering of round-trip SPPs through the nanoaperture (*E*_SPP_, vertical dashed arrows). (**b**) Normalized fluorescence spectra transmitted through the nanohole of a H-3G interferometer with arm length *R*_G_ = 4.05 *μ*m (red line), and through an isolated nanohole serving as reference (black line). The inset shows the corresponding normalized intensity ratio *vs. λ*_em_. (**c**) Fluorescence interferogram obtained by plotting the normalized fluorescence intensity ratio at *λ*_em_ = 750 nm as a function of arm length *R*_G_. The observed oscillation period (*λ*_SPP_/2) is consistent with round-trip SPP interference. (**d**) Color map reporting spectrally resolved fluorescence interferograms *vs*. emission wavelength *λ*_em_ (vertical axis) and interferometer arm length *R*_G_ (horizontal axis).

Figure 1b shows normalized fluorescence spectra transmitted through the hole of a representative interferometer with *R*_G_ = 4.05 *μ*m (*I*_F,n_, red line) and through a reference isolated hole (*I*_F,0,n_, black line), evidencing a remarkable spectral modulation due to SPP-mediated interference (see Methods and Supplementary Fig. S3 for more details on experimental setups and data analysis). The intensity modulation can be quantified by defining a normalized intensity ratio *I*_F,n,r_ = *I*_F,n_/*I*_F,0,n_ *vs*. emission wavelength *λ*_em_, as shown in the inset to Fig. 1b. The normalized intensity modulation at each emission wavelength can also be estimated by measuring the fluorescence spectra through the nanohole of plasmonic interferometers with varying arm length (as reported in Fig. 1c for *λ*_em_ = 750 nm). The fluorescence interferogram oscillates with a period equal to *λ*_SPP_/2, i.e. half the corresponding SPP wavelength, suggesting that the fluorescence modulation is the result of interference from SPPs undergoing a round trip, as reported schematically in Fig. 1a. A color map of the normalized intensity ratio as a function of emission wavelength *λ*_em_ and arm length *R*_G_ is constructed in Fig. 1d by stacking up all of the measured spectra. The color of each pixel in this map corresponds to constructive (red) or destructive (blue) interference, while white pixels correspond to null interference. The 3-groove grating pitch was designed to be half of the SPP wavelength in the three-layer system (silver/Cr^3+^:MgO/air) to keep the reflection by different grooves in phase, thus enhancing the fluorescence modulation up to a factor of ~2. (See Supplementary Figs. S4 and S5 for more details.)

The fluorescence modulation can be totally suppressed if the emitters in the hole are purposely removed, suggesting that the emitters on the planar metal surface play no significant role (see Supplementary Figs. S2 and S6). Therefore, the working hypothesis of this Article is that the fluorescence modification originates solely from interference effects mediated by SPPs that are generated by excited emitters within the nanohole. For this reason, the fluorescence modulation should also be independent of the specific excitation conditions, being only affected by the structural parameters of the interferometer and by changes in the refractive index of the dielectric materials.

### Plasmonic interferometry with incoherent light

**Figure 2:**
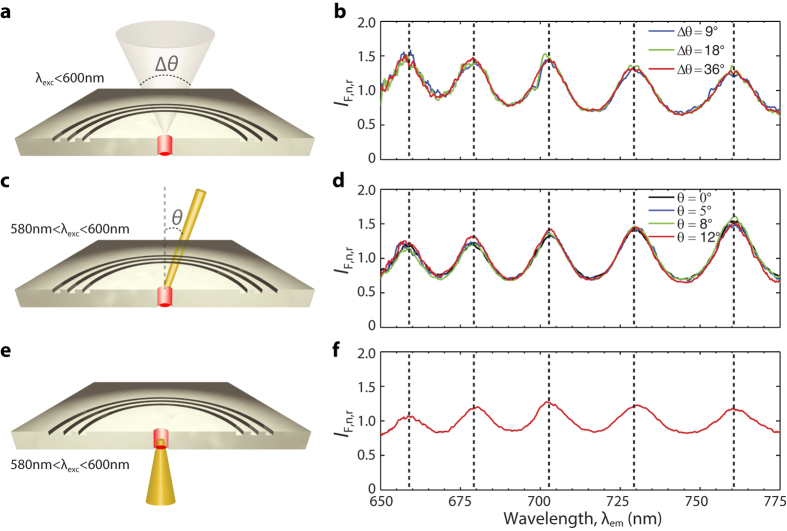
Fluorescence modulation induced by plasmonic interferometry: role of coherence, spectral bandwidth, and incidence angle of the external light source. (**a**) Schematic of a plasmonic interferometer with embedded emitters illuminated by a broadband thermal light source (with excitation wavelengths <600 nm and variable transverse coherence length, achieved by changing the subtended angle Δ*θ*, see Methods). (**b**) Corresponding normalized fluorescence intensity ratios measured at Δ*θ* = 9° (blue), 18° (green), and 36° (red). (**c**) Quasi-monochromatic (580−600 nm) top illumination with varying angle of incidence *θ*. (**d**) Corresponding normalized fluorescence intensity ratios with *θ* = 0° (normal incidence, black), 5° (blue), 8° (green) and 12° (red). (**e**) Quasi-monochromatic (580−600 nm) bottom illumination. (**f**) Corresponding normalized fluorescence intensity ratio. The fluorescence modulation is independent of the specific excitation conditions (coherence, bandwidth, or angle of incidence), as evidenced by the vertical dashed lines in (**b**,**d**,**f**). The arm length of the measured interferometer is 6 *μ*m, the grating pitch is 320 nm.

To demonstrate that coherent excitation is not required to achieve fluorescence modulation, a broadband thermal light source was used to excite the emitters in both a H-3G plasmonic interferometer and a reference isolated hole, using Köhler illumination to tune the degree of spatial coherence^26^ (see Fig. 2a, Supplementary Fig. S7 and Methods). Figure 2b reports normalized fluorescence transmission intensity ratios for various subtended angles Δ*θ* = 9° (blue), 18° (green), and 36° (red), showing no appreciable changes in the observed modulation. Although the emitters are excited by an external light source with low spatial coherence, they retain temporal coherence since the emission occurs from a subwavelength cavity. Indeed, any given photon detected in the far field has to be transmitted through the nanohole and it can originate from two main optical paths, i.e. (1) direct emission from an excited emitter or (2) SPP excitation, round-trip propagation, and diffractive scattering followed by transmission through the same nanoaperture. Since the two paths are indistinguishable due to the subwavelength nature of the aperture, their complex amplitude probabilities (corresponding to the complex field amplitudes *E*_0_ and *E*_SPP_) can be summed up in order to calculate the total far-field fluorescence intensity (as reported in Fig. 1a and Methods). This leads to a coherent interference process that is independent of how the emitter is excited (i.e. either coherently or incoherently) provided the temporal coherence of the emitter is sufficiently high.

To prove that the fluorescence modulation is also independent of precise alignment of the external source, a quasi-monochromatic source (580−600 nm) was used to illuminate the same active plasmonic interferometer with varying angles of incidence (Fig. 2c). Figure 2d shows that the measured normalized fluorescence intensity ratios are also invariant for different incident angles: *θ* = 0° (normal incidence, black), 5° (blue), 8° (green) and 12° (red).

In addition, the measured optical response of the sensor under bottom illumination (Fig. 2e) is shown in Fig. 2f. (See Supplementary Figs. S8 and S9 for more details.) This configuration is especially attractive since for conventional passive plasmonic interferometry the light source and the detector generally need to be placed on opposite sides of the sample^16^,^17^,^18^,^19^,^27^. Moreover, to measure biological samples or liquids, a microfluidic channel is typically required to ensure uniform sample delivery and illumination, thus adding complexity to the sensing architecture. Here we show freedom from that constraint in the novel design, whereby pump and detection can be achieved from the same (i.e. the substrate) side of the sensor. This arrangement does not require a microfluidic channel and solves the common problem arising from potential biological sample damage occurring under direct illumination.

Despite the significantly different illumination conditions, the experimental results in Fig. 2 demonstrate that the optical response of plasmonic interferometers with embedded light emitters is no longer affected by coherence, spectral bandwidth, and incidence angle of the excitation source, thus providing for a more reliable sensing architecture compared to passive interferometry. The spectroscopic capabilities can still be retained by employing a broadband light emitter such as Cr^3+^:MgO.

### Biochemical sensing based on active plasmonic interferometry

**Figure 3:**
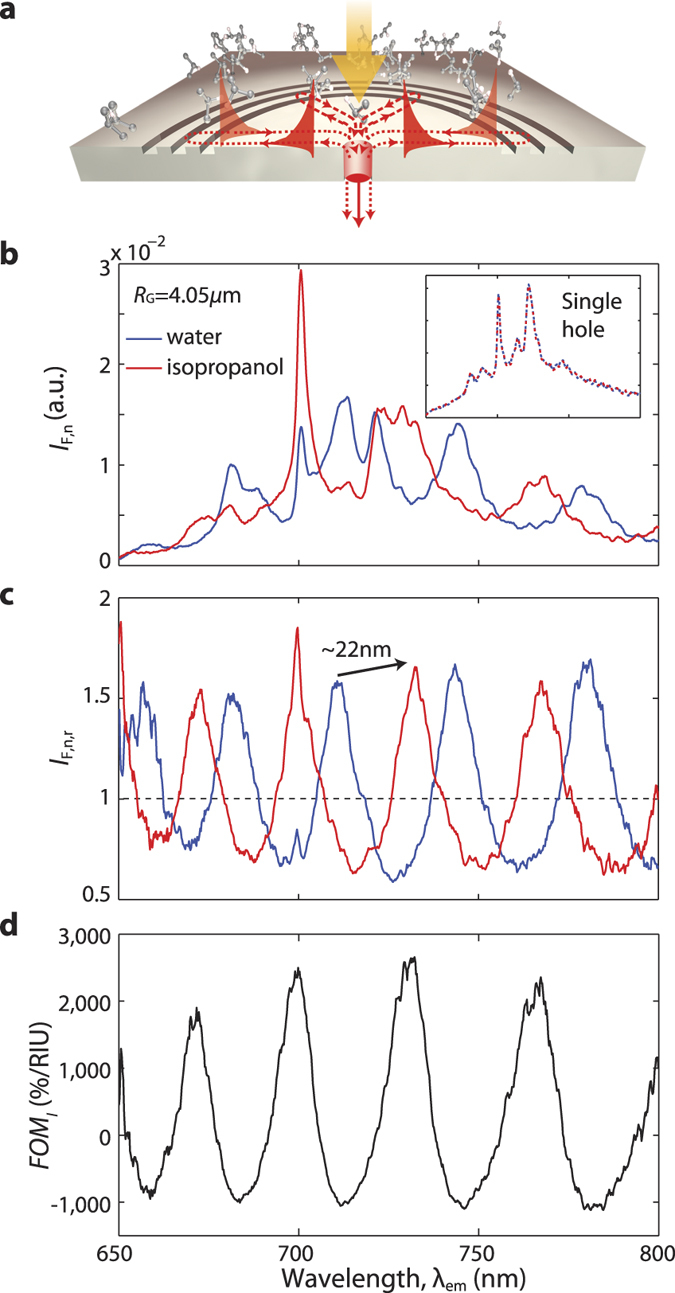
Biochemical sensing experiment in a microfluidic channel: top excitation and bottom detection of fluorescence modulation by plasmonic interferometry. (**a**) Schematic of a circular H-3G plasmonic interferometer with embedded Cr^3+^:MgO emitters under top illumination. Transmitted fluorescence is detected from the bottom side. Molecules of isopropanol are also shown (not to scale). (**b**) Normalized fluorescence spectra transmitted through the nanohole of a circular H-3G plasmonic interferometer with arm length *R*_G_ = 4.05 *μ*m when deionized water (blue) or isopropanol (red) are flown in the microfluidic channel (not shown in **a**). Inset reports the respective reference spectra transmitted through an isolated hole in water (dashed blue) or isopropanol (dashed red). (**c**) Corresponding normalized fluorescence intensity ratios. A clear wavelength shift is observed as a result of the refractive index change. (**d**) Representative figure of merit *FOM*_*I*_ for this specific plasmonic interferometer, calculated according to data in (**c**) and using equation (1).

Figure 3a shows a schematic of a sensing experiment performed under top illumination. Two representative liquids with slightly different refractive index (Δ*n* ≈ 0.05) were delivered onto the metal surface of the active plasmonic interferometers using a microfluidic channel, resulting in a change of the relative SPP optical path length equal to 2*n*_SPP_*R*_G_, see equation (2) in Methods. The pitch of the 3-groove grating in the H-3G plasmonic interferometer was designed to be 230 nm to keep the SPPs reflected by different grooves in phase while flowing the liquid dielectric materials (with *n* ≈ 1.35). (See Supplementary Figs. S10 and S11 for more details.) An additional 10 nm-thick Al_2_O_3_ film was deposited by atomic layer deposition (ALD) to protect the surface of the emitting layer from potential chemical contamination. The inset to Fig. 3b shows the normalized spectra through an isolated single hole in the presence of water (blue line, *n* ≈ 1.33) and isopropanol (red line, *n* ≈ 1.38). No appreciable difference is observed, suggesting that the change of local density of optical states has negligible effects on light emission. In striking contrast, SPP-mediated interference in a H-3G interferometer can determine a dramatic spectral change in the normalized fluorescence spectra and corresponding intensity ratios, as evidenced in Fig. 3b,c, respectively. A figure of merit for this sensing scheme can be defined as follows:


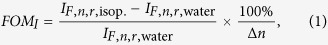


describing the relative intensity change per refractive index unit (RIU). Figure 3d shows the estimated *FOM*_*I*_, with values up to 2,600% RIU^−1^. A ~22 nm red shift is also observed when going from water to isopropanol, with a ~4% refractive index change sufficient to turn constructive interference into destructive, and vice versa (see Fig. 3c). The spectral figure of merit of this sensor can therefore be calculated as 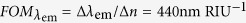.

### Open-surface biochemical sensing on micro-droplets

**Figure 4:**
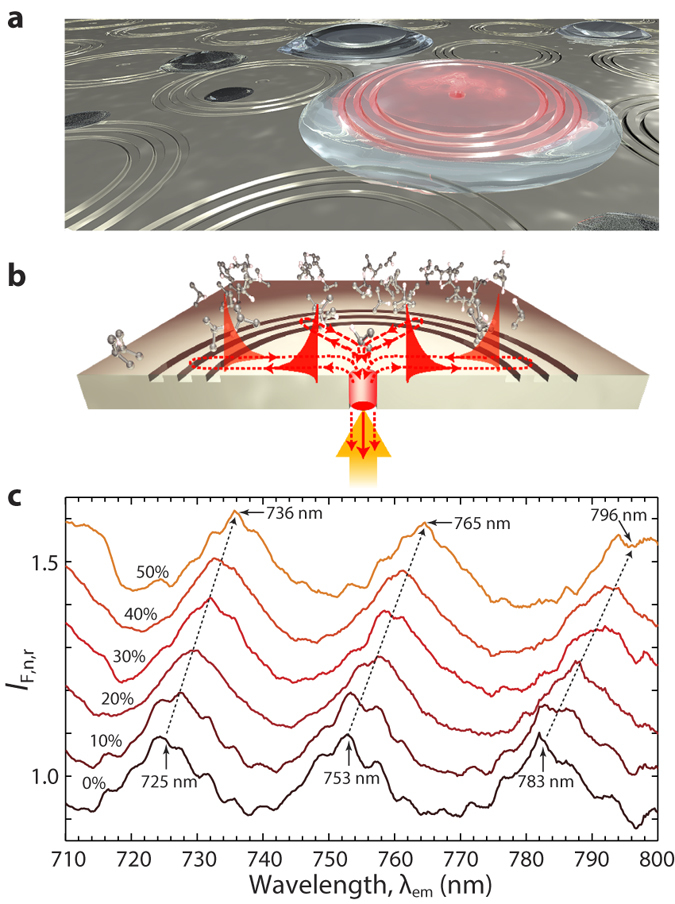
Biochemical sensing experiment on a micro-droplet: same-side (i.e. bottom) excitation and detection of fluorescence modulation by plasmonic interferometry. (**a**) Schematic of a micro-droplet on an array of H-3G plasmonic interferometers with embedded light emitters. Selective detection of the droplet is accomplished by bottom illumination of a specific plasmonic interferometer and detection of the modulated fluorescence (red) transmitted through the nanohole. (**b**) Schematic showing bottom illumination and fluorescence detection for an active H-3G plasmonic interferometer. (**c**) Normalized fluorescence intensity ratios *vs*. *λ*_em_ measured through the nanohole of a H-3G plasmonic interferometer with *R*_G_ = 5 *μ*m, sequentially covered by single micro-droplets with different concentrations of isopropanol: 0% (deionized water), 10%, 20%, 30%, 40%, and 50%. Red shifts of the constructive interference peaks are clearly observed (the dashed lines are guides to the eye). For clarity, the curves corresponding to 10–50% isopropanol have been vertically shifted by a constant offset value, in multiples of 0.1.

To illustrate the advantage of the same-side illumination and fluorescence detection scheme as previously discussed in Fig. 2e,f, we performed sensing experiments by delivering a single micro-droplet of liquid with different concentrations of isopropanol dissolved in water directly on top of an individual H-3G interferometer (with *R*_G_ = 5 *μ*m and 230 nm grating pitch), as schematically shown in Fig. 4a,b. The embedded emitters were illuminated from the bottom side, and fluorescence emission was detected from the same side. Normalized intensity ratios are displayed in Fig. 4c for individual water/isopropanol micro-droplets with varying isopropanol concentrations, from 0 to 50%, in steps of 10% corresponding to a refractive index change of ~0.4%. Clear wavelength shifts are observed, with a red shift of Δ*λ*_*em*_ ≈ 11 nm determined by a refractive index change Δ*n* ≈ 0.025, in agreement with the results reported in Fig. 3. The estimated sensitivity to refractive index change of the proposed sensing scheme (limited by the resolution of our spectrometer) is <5 × 10^−4^, using <1 pL sample volumes.

## Discussion

The performance of the proposed sensing scheme may be limited by the low fluorescence intensity compared with the external pump light and the requirement for spectral analysis as described above. However, such shortcomings are not intrinsic to the proposed platform. For example, by using more efficient light emitters, the fluorescence signal can be greatly enhanced. The light intensity throughput can also be enhanced by integrating a large number of identical interferometers as well as using different configurations (e.g. circular slit-groove plasmonic interferometers that are characterized by a polarization-independent response and higher throughput^27^ compared with the bullseye structure employed in this work). Moreover, by employing shorter wavelength excitation (e.g. ultraviolet light sources), together with bandpass filters or emitters with narrow fluorescence wavelength range, the spectral analysis required here due to the small pump effect can be entirely eliminated.

In summary, we have realized optical interference at the nanoscale with incoherent external light sources. We have accomplished this by implementing solid-state light emitters directly into subwavelength cavities of an array of plasmonic interferometers. The spatial confinement of the emitters results in the generation of coherent SPPs regardless of the coherence state of the external excitation source. The proposed platform enables a novel sensor design that allows measurements in a broader set of environments than previously thought possible. We have demonstrated the efficacy and potential of this alternative sensing scheme in four ways. One is that the fluorescence spectra transmitted through the nanoaperture of each interferometer can be significantly modulated (up to a factor of two) by SPP-mediated interference and used to detect small refractive index changes. Second, in contrast to conventional plasmonic interferometry, the proposed sensing architecture eliminates the need for a coherent, broadband, and precisely aligned external light source, without sacrificing the figures of merit (2,600% RIU^−1^ and 440 nm RIU^−1^). Third, such a sensing scheme enables same-side excitation and detection of fluorescence modulation which allows for direct exposure of the top surface to the analyte of interest. This eliminates the need for a microfluidic channel to control sample flow, and addresses the problem of misalignment induced by lensing effects arising from the droplet surface curvature. And fourth, the proposed sensing technology can be further extended to include various light emitters other than Cr^3+^:MgO, such as semiconductor quantum dots^28^,^29^,^30^ (see Supplementary Fig. S12), as well as electrically excited emitters^31^,^32^,^33^ to realize fully integrated, electrically-driven biochemical sensors.

## Methods

### Fabrication

A 300 nm-thick silver film was deposited on a quartz substrate previously coated with a 4 nm-thick titanium adhesion layer using electron-beam evaporation. Then, circular hole-3grooves (H-3G) plasmonic interferometers were fabricated on the silver film using focused ion beam (FIB) milling, together with isolated holes as references. Following the FIB milling, a ~40 nm-thick Cr^3+^:MgO (1 wt%) emitter layer was deposited on the chip surface using electron-beam evaporation. Each H-3G interferometer consists of a ~300 nm-diameter hole and three ~200 nm-wide, ~40 nm-deep concentric grooves. The radius of the inner groove was varied from 0.25 *μ*m to 6 *μ*m in steps of 25 nm (231 distinct devices in total), and the pitch for the grooves was designed to be 320 nm to keep the reflected SPP in phase for propagation at the silver/air interface. All the isolated holes are identical to the corresponding holes in H-3G interferometers. See Supplementary Fig. S1 for more details on various kinds of plasmonic interferometers also explored in this study. For the biochemical sensing experiments, another chip containing active plasmonic interferometers was fabricated with an additional thin (10 nm) Al_2_O_3_ layer deposited on top of Cr^3+^:MgO using atomic layer deposition (ALD) at low temperature (75 °C) to protect the sensor surface and prevent direct contact of the emitting layer with the liquid samples, thus eliminating chemical contamination. Note that in this case (i.e. silver/40 nm Cr^3+^:MgO/10 nm Al_2_O_3_/liquid) the groove pitch was designed to be 230 nm to keep the reflected SPP in phase while propagating at the silver/liquid interface (*n* ≈ 1.35, see Supplementary Figs. S10 and S11).

### Experimental setup

Although in this Article we demonstrate that the sensing scheme is unaffected by the specific light source and illumination conditions, to perform the experiments we used a conventional Xenon arc lamp and a supercontinuum laser, coupled to an optical microscope and spectrograph to detect fluorescence spectra. In particular, the data shown in Figs. 1, 2d and 3 were acquired by illuminating the plasmonic interferometers with a quasi-monochromatic (590 ± 10 nm) light beam from a supercontinuum laser (NKT Photonics, SuperK Extreme) using a wavelength selector (NKT Photonics, SuperK Varia) and then focused on the fabricated plasmonic interferometers by the microscope condenser lens to excite the emitters (top illumination). An inverted microscope (Nikon, Eclipse Ti) with a 40× and 0.6 NA objective was used to collect the fluorescence spectrum transmitted through each nanohole (either individual, or as part of the plasmonic interferometer), which was then projected onto the entrance slit of a spectrograph (Princeton Instruments, Acton SpectraPro SP-2300) and dispersed on a CCD camera (Princeton Instruments, Pixis 100) by a diffraction grating optimized for the specific detection wavelength range. An automated stage was used to scan and acquire the corresponding spectra from all the H-3G/hole nanostructures. Note that active fluorescence under bottom illumination (shown in Figs. 2e,f and 4) was acquired through the same 40×, 0.6 NA objective, used for both excitation and collection of the fluorescence emission. The broadband thermal light source used in Fig. 2a,b was a Xenon arc lamp combined with a 600 nm short pass filter. The transverse spatial coherence was tuned by employing Köhler illumination^26^. Specifically, by increasing the diameter of the microscope condenser aperture diaphragm, the transverse wavevector of the incident light can be made to span a broader range, which in turn determines a decrease in the spatial coherence at the hole location^26^. A microfluidic channel made by polydimethylsiloxane (PDMS) was used to deliver and control the flow of water and isopropanol onto the plasmonic interferometer to obtain the results shown in Fig. 3. For the sensing experiments reported in Fig. 4, liquid droplets containing various concentrations of water and isopropanol were generated using a micro-pipette dispenser.

### Data analysis

The fundamental optical properties of plasmonic interferometers can be captured by the ratio (*I*_F,r_) between the measured fluorescence transmission intensity through the nanoaperture of a plasmonic interferometer (*I*_F_) and the reference fluorescence intensity transmitted through an isolated identical nanoaperture (*I*_F,0_):


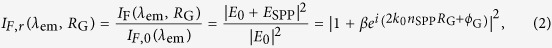


where the subscript F indicates active fluorescence transmission, *E*_0_ and *E*_SPP_ are the complex electric field amplitudes of the direct emission and SPP-induced fluorescence transmitted through the nanoaperture, respectively. *k*_0_ = 2*π*/*λ*_em_ is the amplitude of the free-space wave vector, *λ*_em_ is the free-space emission wavelength, *R*_G_ is the interferometer arm length, defined as the inner circular groove radius (see Fig. 1a), *β* is a wavelength-dependent effective SPP scattering coefficient defined with respect to the directly downward emission, which also includes the in-plane reflection coefficient of the groove, *n*_SPP_ = *λ*_em_/*λ*_SPP_ is the complex, wavelength-dependent SPP refractive index in the three-layer system, *ϕ*_G_ is the wavelength-dependent phase change induced by the scattering processes, and the constant 2 in the argument accounts for the SPP round trip.

In order to eliminate any additional (although weak) excitation effects due to the direct modulation of the pump intensity caused by the plasmonic interferometer (see Supplementary Fig. S3), the fluorescence spectra were first normalized to their integrated intensity. The ratio of the normalized spectra can then be calculated as:


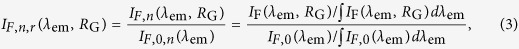


where *I*_F,n_ and *I*_F,0,n_ are the normalized fluorescence spectra transmitted through the nanoaperture of an interferometer and through a reference isolated aperture, respectively. Combining equations (2) and (3), *I*_F,n,r_ can also be expressed as:


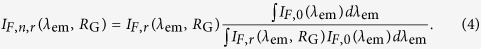


Equation (4) was used to model the normalized fluorescence intensity ratio color map (Supplementary Fig. S5). Note that equation (2) is valid only when the SPP generation process represents a coherent alternative path for the excited emitters in the nanoaperture. The agreement between simulations and experiments (see Supplementary Fig. S5) suggests that the degree of coherence of the emitters in the nanoaperture is sufficiently high to justify the use of equation (2). This is further supported by the fact that no appreciable attenuation is observed in the fluorescence intensity amplitude modulation within the range of interferometer arm lengths used in this experiment.

## Additional Information

**How to cite this article**: Li, D. *et al.* Nanoscale optical interferometry with incoherent light. *Sci. Rep.* **6**, 20836; doi: 10.1038/srep20836 (2016).

## Supplementary Material

Supplementary Information
