# Supplementary material for: Nanoscale optical interferometry with incoherent light
Source: Sci Rep. 2016 Feb 16;6:20836. doi: 10.1038/srep20836 (PMC4754639; doi:10.1038/srep20836)
Supplement: Supplementary Information [file srep20836-s1.pdf]

Nanoscale optical interferometry with incoherent light

Dongfang Li, Jing Feng, and Domenico Pacifici*
School of Engineering, Brown University, Providence, Rhode Island, 02912, United States

List of Supplementary Figures

1. Fig. S1: Scanning electron microscope (SEM) images of representative slit-groove (SG), hole-1groove (H-1G), hole-2groove (H-2G), and hole-3groove (H-3G) plasmonic interferometers with varying arm length.

2. Fig. S2: Mechanism of SPP excitation by emitters embedded in active plasmonic interferometers.

3. Fig. S3: Quantification of weak pump effects on fluorescence intensity caused by modulation of the excitation beam by SPP-mediated interference in plasmonic interferometers.

4. Fig. S4: Fluorescence spectra transmitted through the nanoapertures of slit-groove (SG) and hole-Ngroove (H-NG) plasmonic interferometers.

5. Fig. S5: Theoretical fits of transmitted fluorescence spectra for active hole-1groove (H-1G) plasmonic interferometers.

6. Fig. S6: Suppressed fluorescence intensity modulation in the absence of emitters within the central nanohole of plasmonic interferometers.

7. Fig. S7: Spectral comparison of the broadband white light and fluorescence.

8. Fig. S8: Color maps of normalized fluorescence intensity ratios transmitted through the nanoapertures of H-1G, H-2G, and H-3G plasmonic interferometers: comparison between top and bottom illumination.

9. Fig. S9: Comparison between normalized fluorescence spectra transmitted through an individual nanohole under top and bottom illumination.

10. Fig. S10: Biochemical sensing experiments employing hole-Ngroove (H-NG) plasmonic interferometers with embedded light emitters, with $N = 1, 2,$ and 3, using various liquids.

11. Fig. S11: Comparison between biochemical sensing experiments for different values of groove number and grating pitch.

12. Fig. S12: Color maps of normalized fluorescence intensity transmitted through the nanoslit of slit-groove and groove-slit-groove plasmonic interferometers coated with a ∼20nm-thick layer of CdSe quantum dots.

*Electronic address: `Domenico_Pacifici@brown.edu`

I Fabrication of plasmonic interferometers

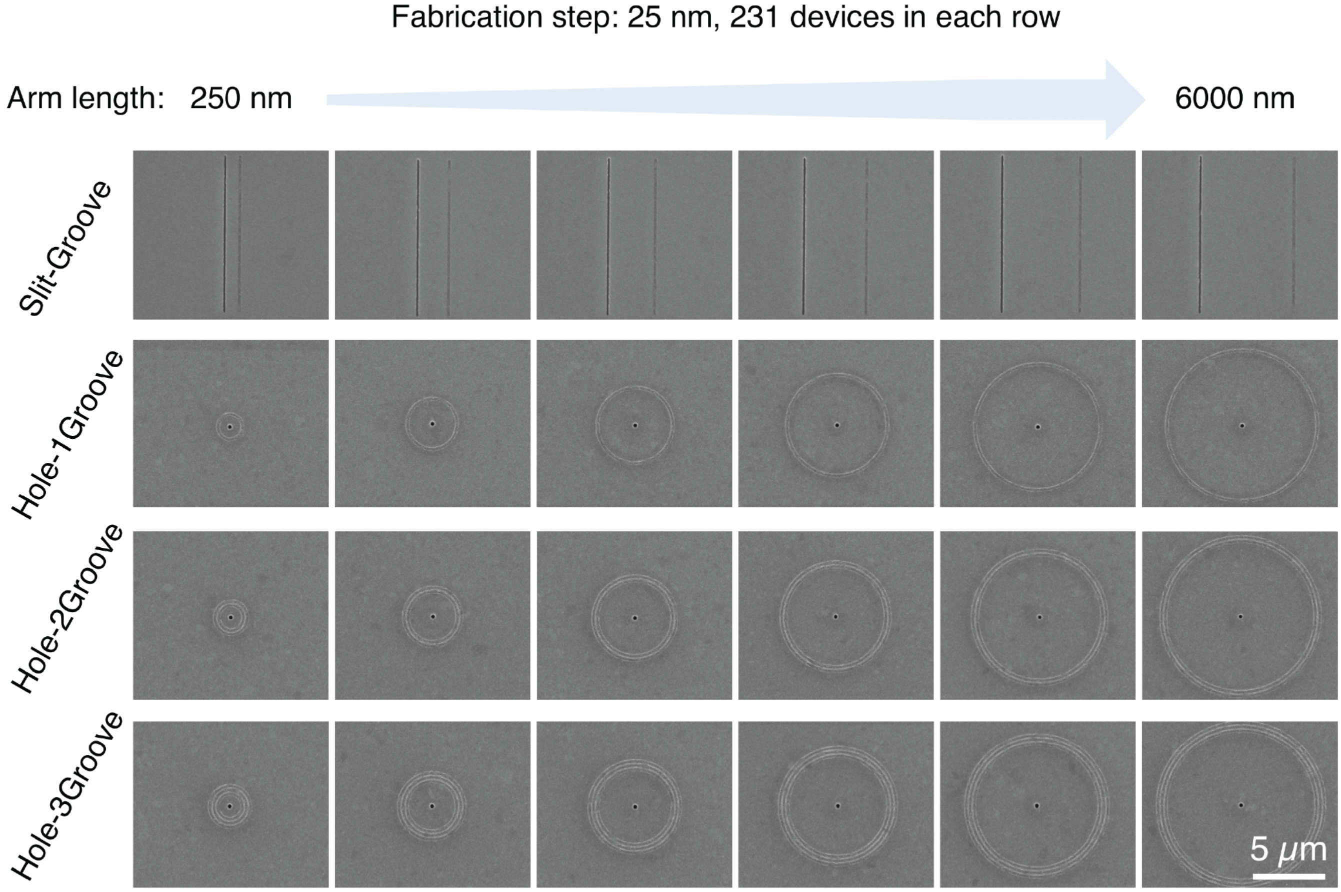

FIG. S1: **Fluorescence spectra transmitted through the nanoapertures of slit-groove (SG) and hole-Ngroove (H-NG) plasmonic interferometers.** Four kinds of plasmonic interferometers were studied in this Article, consisting of slit-groove (SG), hole-1groove (H-1G), hole-2groove (H-2G) and hole-3groove (H-3G) structures, although results for just H-3G plasmonic interferometers were reported in the main text since these structures show strongest spectral modulation. The arm lengths (i.e., the separation between slit and groove, d_{SG}, or the inner circular groove radius, R_{G}), was increased from 250 nm to 6000 nm in steps of 25 nm. For each kind of plasmonic interferometer, 231 distinct devices were fabricated and their optical properties were investigated (see Fig. S4 for a comparison of main results). Note that the distance of the adjacent devices in each row (as shown above) is 25 μm, while the distance of the adjacent rows is 200 μm. For the H-NG sample, 3 rows of single holes, 3 rows of H-1G, 1 row of H-2G and 1 row of H-3G are fabricated, which means the measurement area is around 1.4×6 mm^2. The smooth data shown in Fig. S4 confirm the uniformity of the deposited films.

II Mechanism of SPP excitation by emitters embedded in active plasmonic interferometers

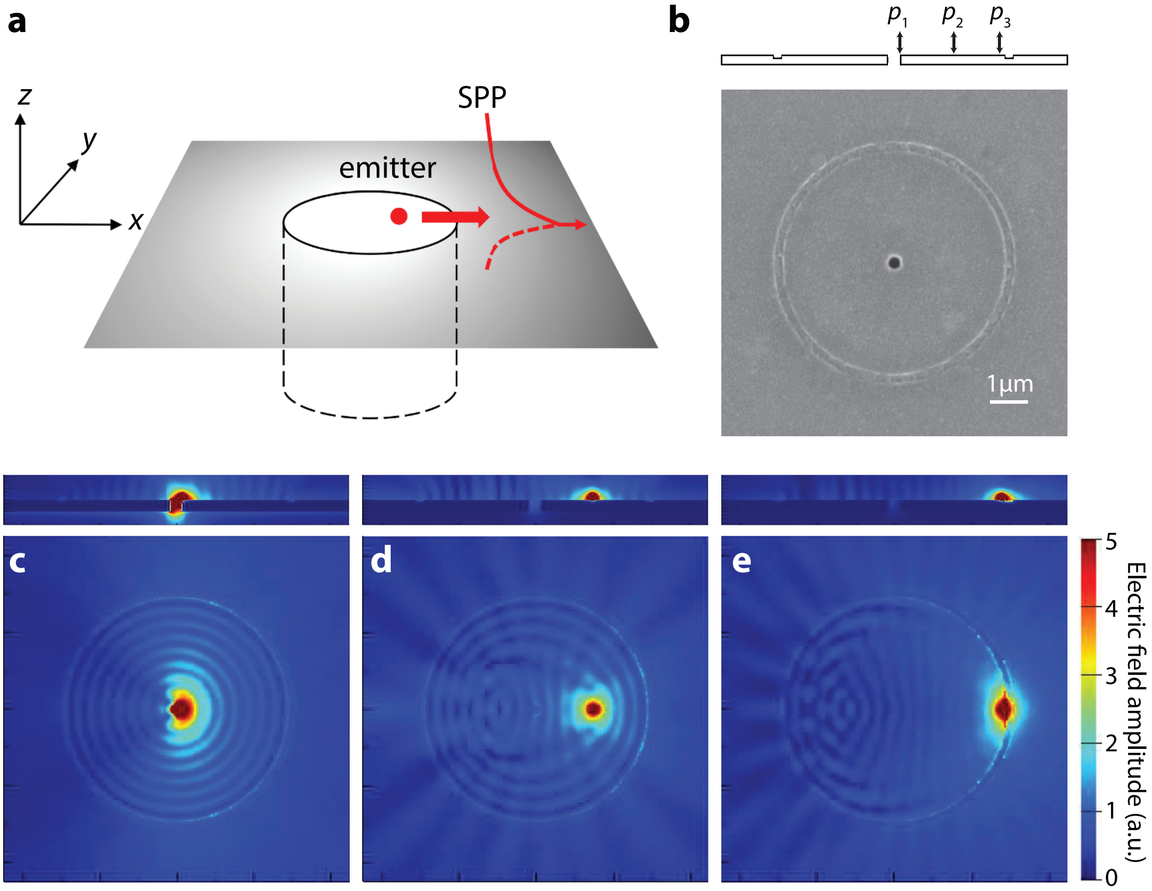

FIG. S2: **Mechanism of SPP excitation by emitters embedded in active plasmonic interferometers.** **a**, The schematic shows the generation of SPPs (red curve) by non-radiative decay of an excited emitter (red dot) in a nanohole. The cylinder represents the nanohole and the gray plane indicates the top surface of the metal film. **b**, SEM image of a hole-1groove plasmonic interferometer with $R_G = 3$ μm and the corresponding cross section in x-z plane. p_1, p_2, p_3 indicate z-dipoles placed in different distances away from the central hole. Note that only the representative results of z-dipoles simulated by the finite difference time domain (FDTD, Lumerical Solutions Inc.) are shown here, since the emission of z-dipole strongly contributes to the fluorescence transmission through the nanohole compared with x- and y-dipoles. **c-e** show the amplitude of electric field at the cross sections in x-y plane (bottom panels, 9×9 μm^2 area) and x-z plane (top panels, 1.3×9 μm^2 area) for z-dipoles placed in 0.15 μm, 1.5 μm and 2.8 μm away from the central hole and 20 nm above the silver film. The standing wave is clearly evidenced and the contribution of dipoles far away from the central hole is much smaller than those near the hole. This, together with the experimental results shown in Fig. S6, further supports our hypothesis that the fluorescence modification is only from interference effects mediated by SPPs generated by excited emitters in/nearby the nanohole. Note that the electric field for the bottom panels of **c-e** are taken from the monitor 20 nm above the silver film. In the simulation, the emission wavelength is 725 nm (i.e., central emission wavelength of Cr^{3+}:MgO), the silver film thickness is 300 nm, the groove is 200nm wide and 50nm deep.

III Weak pump effects on the modulation of fluorescence intensity

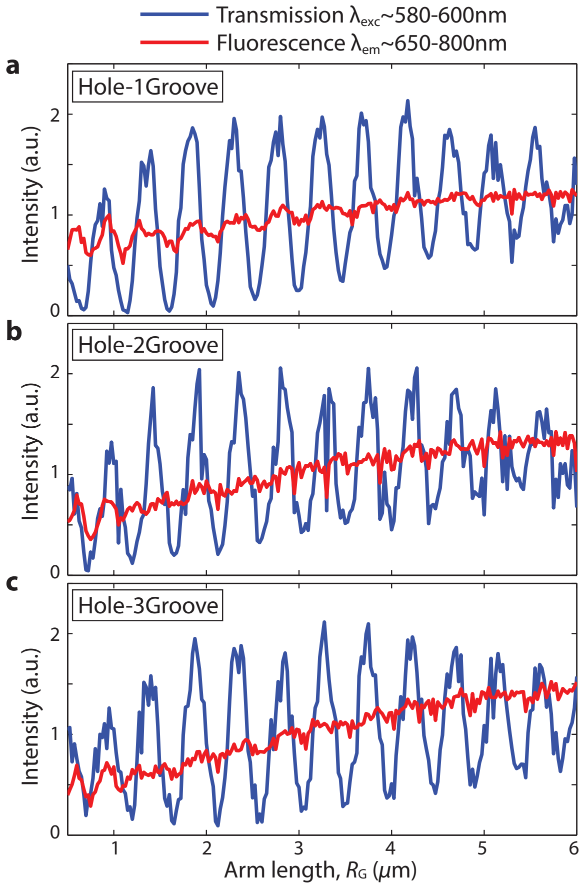

FIG. S3: **Quantification of weak pump effects on fluorescence intensity caused by modulation of the excitation beam by SPP-mediated interference in plasmonic interferometers. a–c**, The blue lines indicate the total transmission intensity integrated over the excitation wavelength range (580 to 600 nm) as measured in the passive transmission scheme (at normal incidence), while red lines represent the integrated fluorescence intensity transmitted through the apertures of hole-Ngroove plasmonic interferometers. The total transmitted fluorescence intensity is slightly modulated in phase with the constructive or destructive interference conditions for the excitation pump at the nanoaperture position, as a function of arm length. In order to account for and remove this weak modulation effect, we normalized each fluorescence spectrum to its corresponding integrated intensity, as reported in the Methods section of the main text. It is worth noting that, although generally small, such pump effects can be entirely suppressed by using an ultraviolet light source to pump the emitters. In this case the SPPs excited by the external light source will decay quickly away from the nanostructure thus making the spectral normalization process no longer necessary.

IV Fluorescence modulation for slit-groove (SG) and hole-Ngroove (H-NG) plasmonic interferometers: a comparative study

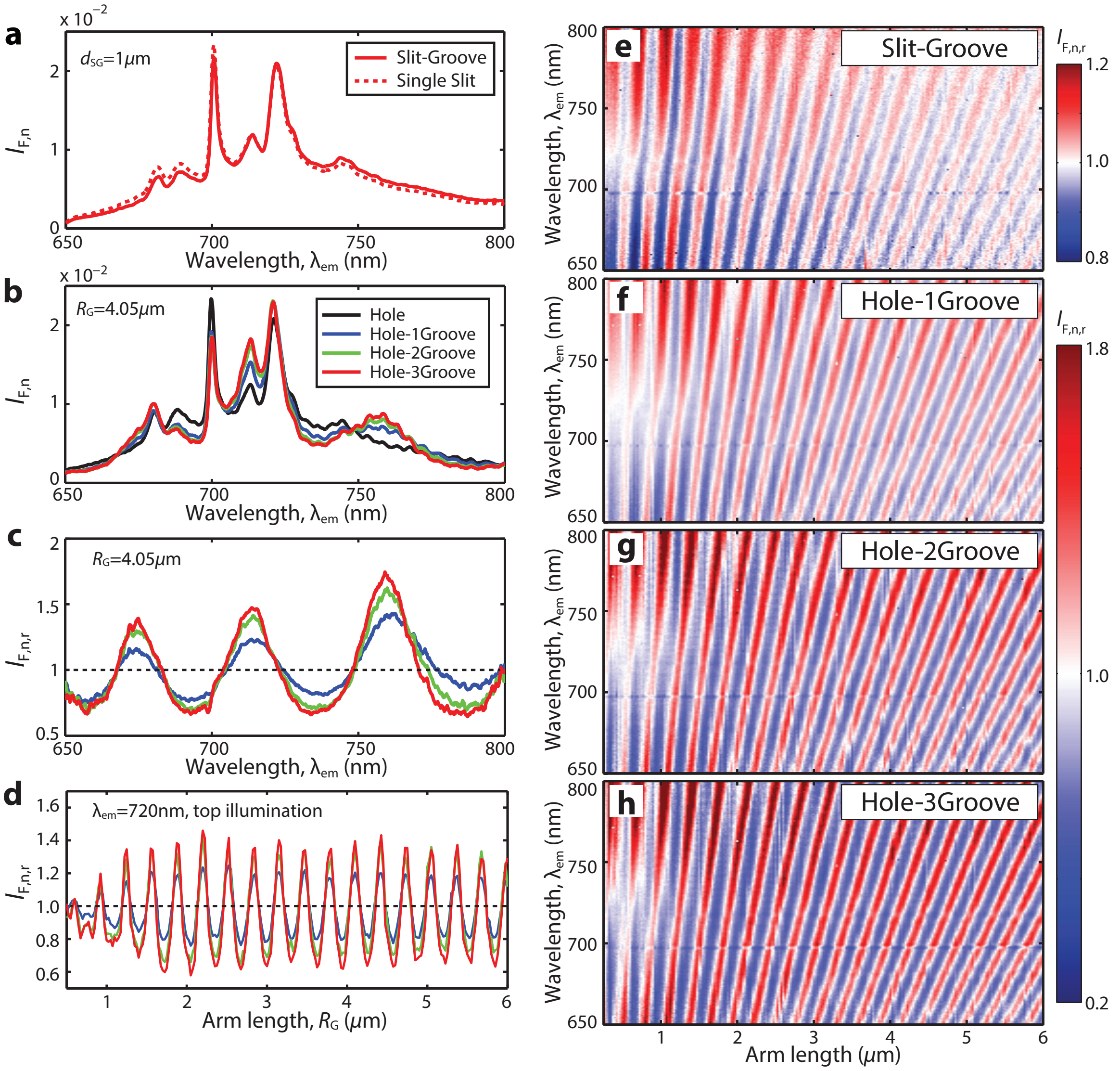

FIG. S4: **Fluorescence spectra transmitted through the nanoapertures of SG and H-NG plasmonic interferometers.** **a**, Active transmission spectra for a representative SG plasmonic interferometer with arm length $d_{\mathrm{SG}} = 1\ \mu$m (solid red line), together with transmission spectra through a reference individual slit (dashed red line). **b**, Normalized fluorescence intensity spectra through a single reference hole (black) and H-NG plasmonic interferometers (blue, $N = 1$; green, $N = 2$; and red, $N = 3$, respectively) at a fixed interferometer arm length $R_{\mathrm{G}} = 4.05\ \mu$m. **c**, Normalized fluorescence intensity ratio obtained by dividing the normalized fluorescence transmission spectra through the hole of each plasmonic interferometer in **b** by the single-hole reference spectrum. **d**, Normalized fluorescence intensity ratios of H-NG interferometer at $\lambda_{\mathrm{em}} = 720$ nm for top illumination, *vs.* interferometer arm length R_{G}. **e-h**, Color maps of normalized transmission fluorescence intensity ratios for SG, H-1G, H-2G and H-3G plasmonic interferometers measured under top illumination (excitation wavelength spanning the range 580-600 nm). Note that the pitch for the $N = 2, 3$ grooves (320 nm) was chosen to maintain the phase relationship between the in-plane SPPs reflected by each individual groove. The SPP-mediated interference and fluorescence intensity modulation depth were increased by adding extra grooves at optimized distances (**f-h**).

V Theoretical fits of fluorescence modulation for active hole-1groove (H-1G) plasmonic interferomters with embedded light emitters

At any given wavelength, equations (2) and (4) described in the Methods section of the main text can be used to fit the experimental normalized intensity ratio (i.e., horizontal cut in Fig. S5a) as a function of R_G, with β and ϕ_G as the fitting parameters. The fit can be extended to all wavelengths to generate Fig. S5b, that shows good agreement with the experimental map (Fig. S5a). In the fitting process, the complex refractive index of SPPs was calculated numerically using a three-layer system (silver/ 40nm Cr^{3+}:MgO/air) by employing the finite difference frequency domain (FDFD) method [1], where the refractive index ($n_d \approx 1.66$ at the fluorescence wavelength range of interest) and the thickness (40 nm) of the Cr^{3+}:MgO active layer was directly measured by variable angle spectroscopic ellipsometry (VASE) while the silver refractive index was calculated by using the Drude-Lorentz model [2]. The agreement is further evidenced by a direct comparison between experimental (red dots) and theoretical (blue line) cross sections at λ_{em} = 720 nm (Fig. S5c), which shows ∼20% intensity modulation efficiency. The fluorescence spectrum (i.e., corresponding to a vertical cut in Fig. S5a) of a specific H-1G interferometer ($R_G = R_{G0}$) can also be reconstructed by using the expression $I_{F,n}(\lambda_{em}, R_{G0}) = I_{F,n,r}(\lambda_{em}, R_{G0}) I_{F,0,n}(\lambda_{em})$, where $I_{F,n,r}$ is obtained by fits and $I_{F,0,n}$ is experimentally measured. The fluorescence spectra for a representative H-1G interferometer with R_G = 4.05 μm (solid red) and the theoretical prediction (dashed blue) are reported in Fig. S5d, together with that of a single hole (solid black), showing again good agreement. These results provide strong evidence and support to the SPP-mediated round trip interference effect in the observed fluorescence spectra modulation (as represented schematically in the main text, Fig. 1a).

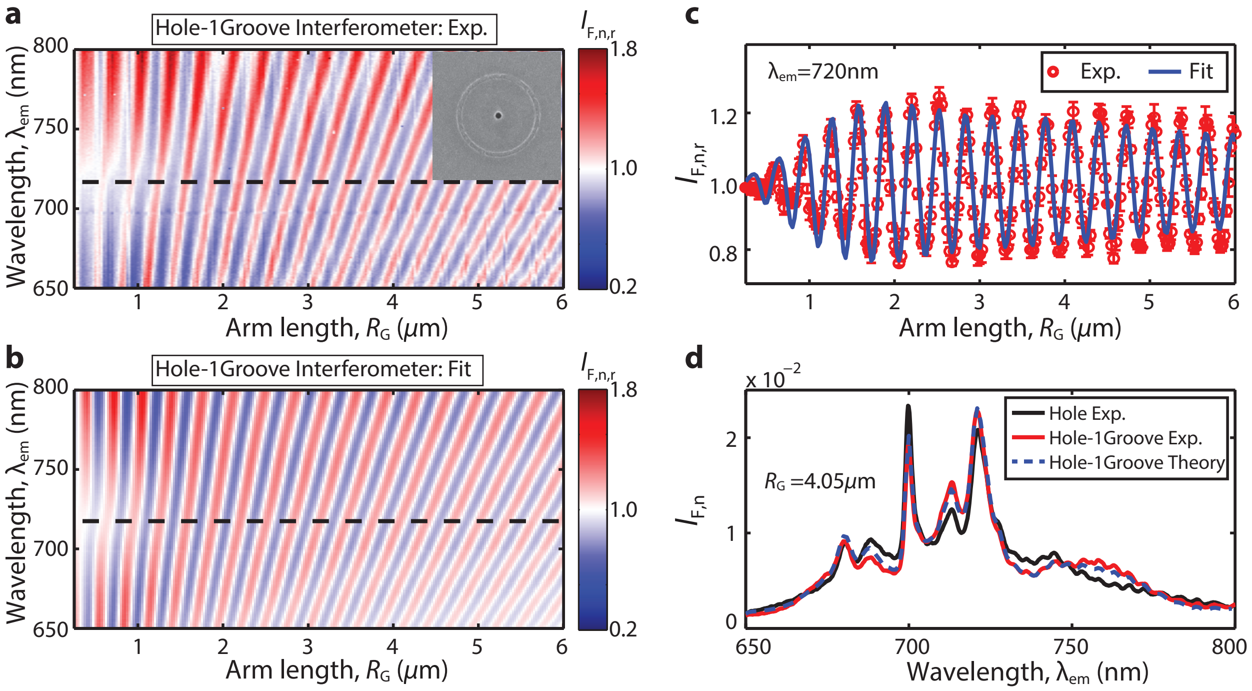

FIG. S5: **Theoretical fits of transmitted fluorescence spectra for active hole-1groove (H-1G) plasmonic interferometers.** **a**, Color map reporting the normalized Cr^{3+}:MgO fluorescence intensity ratio transmitted through the nanohole of circular H-1G plasmonic interferometers as a function of interferometer arm length R_G and emission wavelength λ_{em}. **b**, Simulated color map obtained by fitting the data reported in **a** using equations (2) and (4). **c**, Experimental normalized intensity ratio (red dots) and corresponding theoretical fit (blue line) at λ_{em}=720 nm, indicated by the black dashed lines (i.e., horizontal cut) in **a**,**b**. The error bars at each arm length are calculated taking into account propagation of experimental errors and statistical variance obtained by performing the experiments on three plasmonic interferometers with identical arm length. **d**, Experimental (solid red) and theoretical (dashed blue) normalized fluorescence spectra for a circular H-1G interferometer with R_G = 4.05 μm; the measured fluorescence spectrum through an isolated reference hole is also shown (solid black).

VI Transmitted fluorescence spectra for hole-1groove (H-1G) plasmonic interferometers with and without emitters inside the nanoaperture

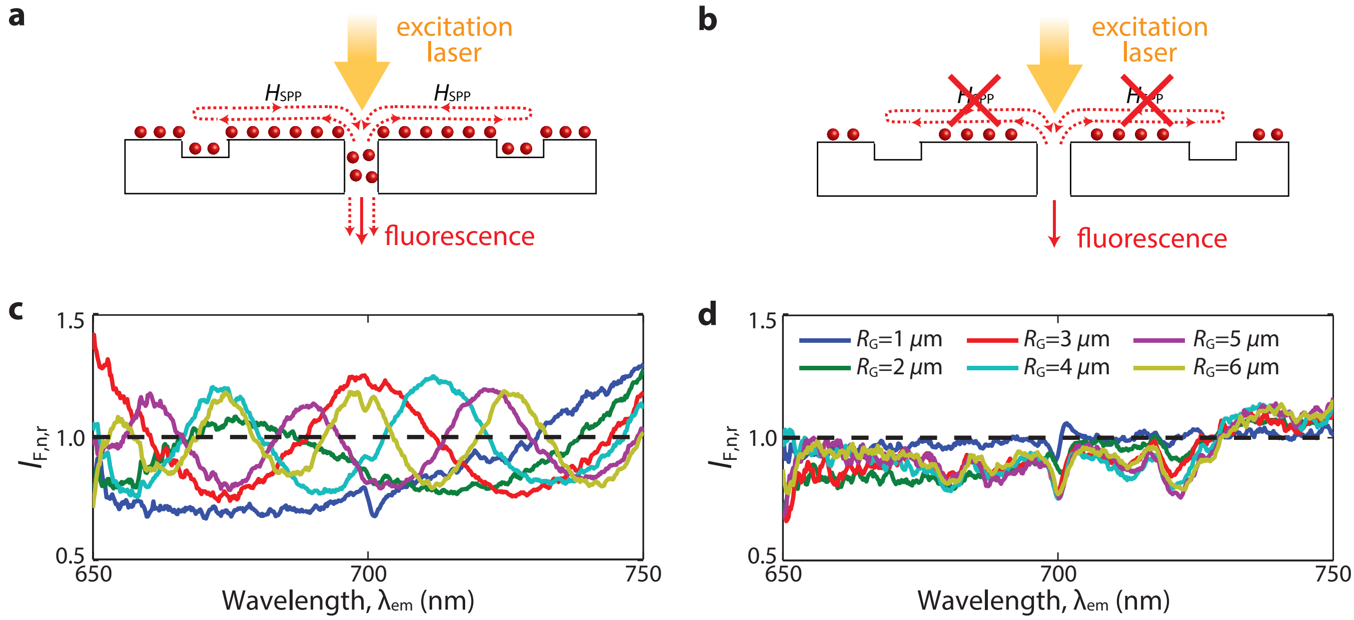

FIG. S6: **Suppressed fluorescence intensity modulation in the absence of emitters within the central nanohole of plasmonic interferometers. a,** Schematic of H-1G plasmonic interferometer with emitters embedded in the subwavelength aperture (i.e., the interferometer was fabricated before depositing the emitting layer on top of the metal surface, so that the emitters would fill the central hole volume). **b,** Schematic of H-1G plasmonic interferometer without emitters (i.e., the interferometer was fabricated after deposition of the emitting layer to etch away the light emitters from the central aperture volume with FIB milling). **c** and **d** show the normalized active fluorescence intensity ratio for the pre- and post-fabricated H-1G interferometers (i.e., with and without emitters inside the central hole). No fluorescence spectral oscillations are observed for the post-fabricated structures, which supports our assumption that the fluorescence from just the emitters inside and/or in the vicinity of the nanoaperture can be strongly modified, while the spectral modulation of emitters away from the nanoaperture is averaged out to zero, or is negligible.

VII Spectral comparison of the broadband white light and fluorescence

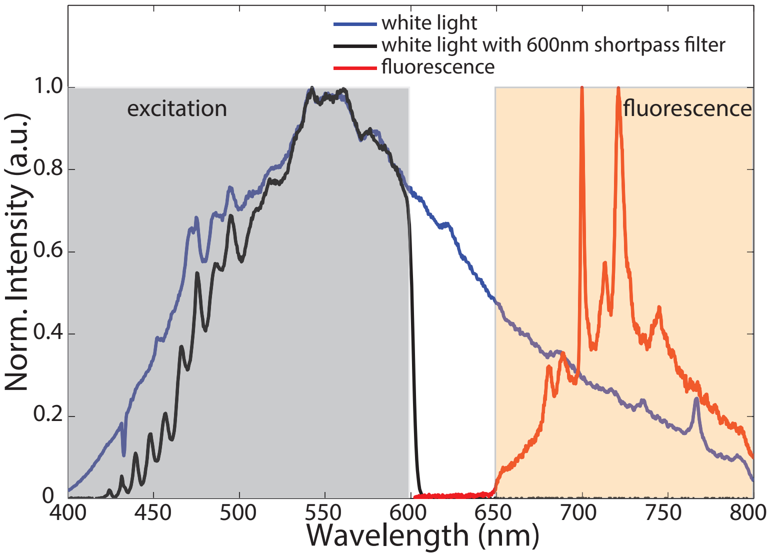

FIG. S7: **Spectral comparison of the broadband white light and fluorescence through a single hole.** The blue curve represents the spectrum of the Xenon arc lamp without any filter. The black curve is the spectrum of the Xenon arc lamp measured with a 600 nm shortpass filter, which is used to illuminate the sample in Fig. 2a of the main text. The red curve shows the fluorescence spectrum measured with a 650 nm longpass filter. The blue shaded area stands for excitation wavelength range while the red shaded area denotes the fluorescence wavelength range. Note that all the spectra were measured with the subtended angle $\Delta\theta = 9^\circ$ of the illuminating white light.

VIII Fluorescence modulation through the nanohole of circular hole-Ngroove plasmonic interferometers under bottom illumination

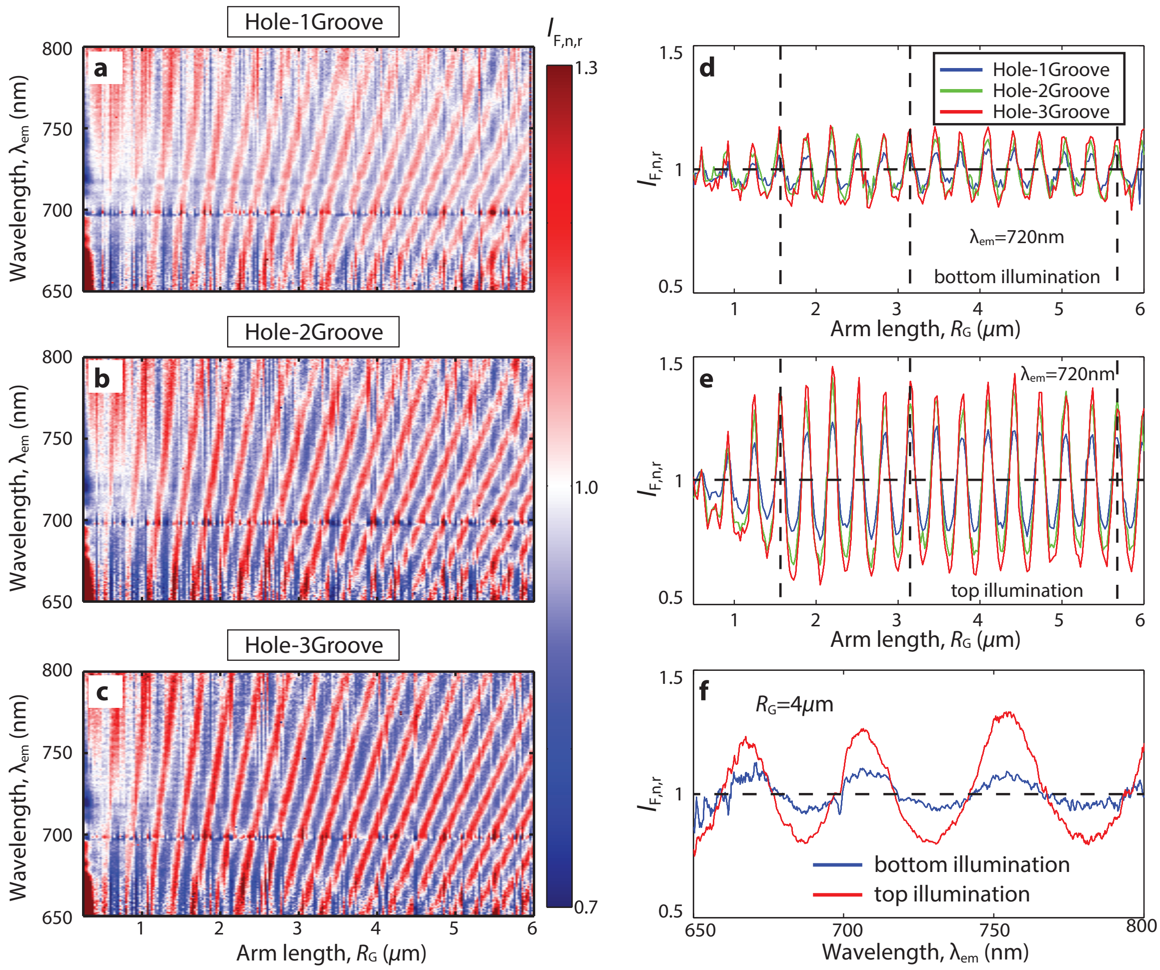

FIG. S8: **Comparison between normalized fluorescence spectra transmitted through an individual nanohole under top and bottom illumination. a-c** show the normalized fluorescence intensity ratio color maps for H-1G, H-2G and H-3G plasmonic interferometers under bottom illumination. Despite the intensity modulation depth is slightly reduced, the positions of the red and blue bands in the color maps match very well those obtained under top illumination (Fig. S4f-h), as indicated by vertical dashed lines in **d** and **e**. The weaker intensity modulation depth under bottom illumination can be explained by considering that the emitters directly sitting on top of the quartz substrate right underneath the bottom mouth of the nanoaperture are more efficiently pumped when bottom illumination is employed (see Fig. S9), thus reducing the overall SPP coupling efficiency. **d** and **e** show the normalized intensity ratio as a function of arm length R_G (one-, two- and three-groove are shown in blue, green and red, respectively) at λ_{em} = 720 nm for bottom (**d**) and top (**e**) illumination conditions, respectively. **f**, Normalized intensity as a function of wavelength at R_G=4 μm for H-1G plasmonic interferometer under top (red) and bottom (blue) illumination. The resonant wavelengths match well with each other, suggesting that the SPP round-trip effect is insensitive to excitation conditions.

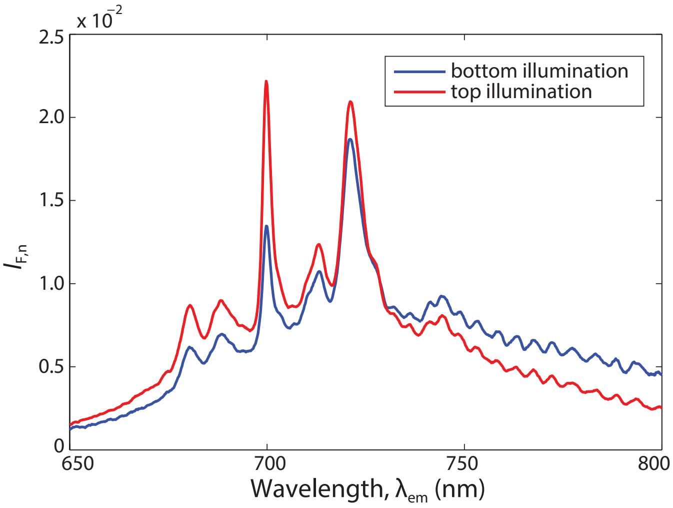

FIG. S9: **Comparison between normalized fluorescence spectra transmitted through an individual nanohole under top and bottom illumination.** Two representative Cr^{3+}:MgO emission spectra transmitted through the same individual hole under different, i.e. bottom (blue) and top (red), illumination conditions. As reported by Karaveli *et al.*[3], the zeroth phonon line (ZPL) emission at 700 nm has magnetic nature. The suppression of this peak under bottom illumination is determined by a higher excitation efficiency of the emitters that are in closer contact with the quartz substrate. This also justifies the observed spectral change through the same individual nanohole. However, the coupling efficiency to surface plasmon polaritons for these emitters is weaker, which explains the slightly reduced spectral modulation depth observed under bottom illumination (Fig. S8).

IX Biochemical sensing experiments: role of groove number and grating pitch

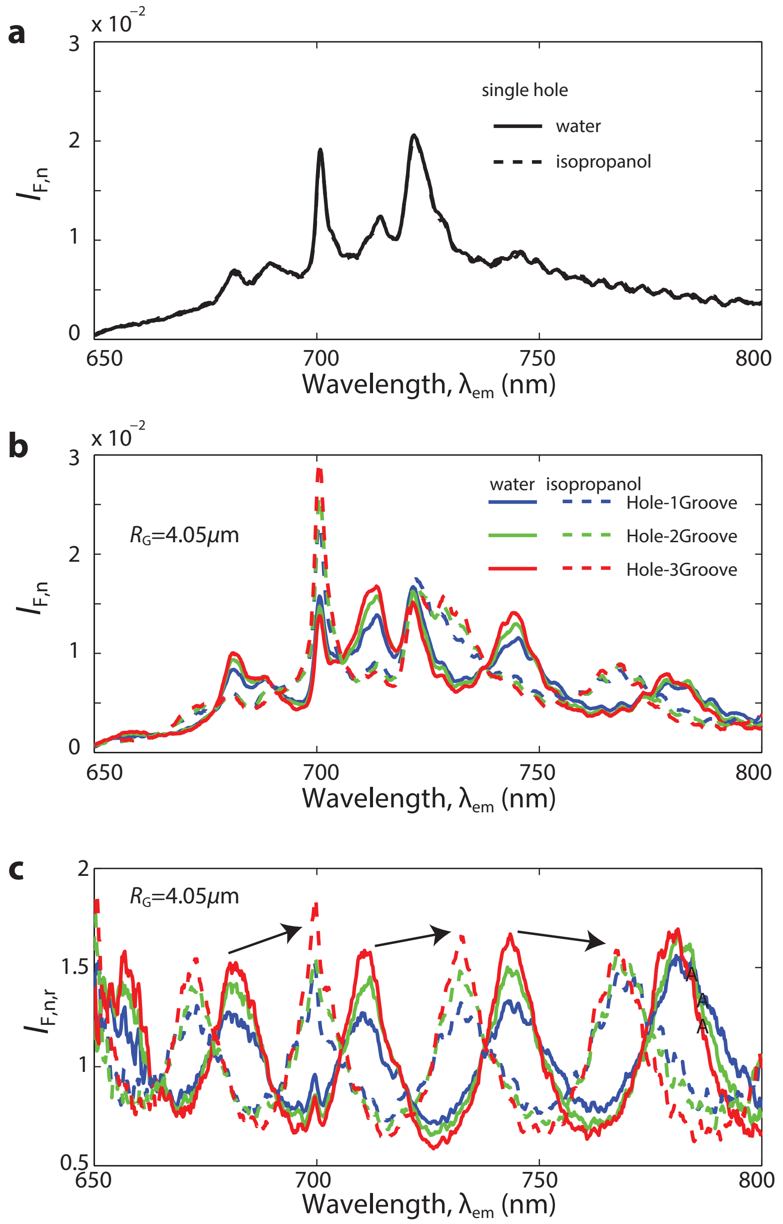

FIG. S10: **Biochemical sensing experiments employing hole-Ngroove (H-NG) plasmonic interferometers with embedded light emitters, with $N = 1, 2$, and 3, using various liquids.** **a**, Normalized fluorescence intensity spectra measured through a single nanohole while flowing water (solid line) or isopropanol (dashed lines), respectively. No significant differences were observed, suggesting that the change in local density of optical states due to the slight refractive index change is not strong enough to modify the fluorescence spectra. **b**, Normalized fluorescence intensity spectra measured through the central nanohole of H-NG plasmonic interferometers (blue $N = 1$, green $N = 2$, and red $N = 3$) with inner groove radius $R_G = 4.05$ μm and 230nm grating pitch, for water (solid lines) and isopropanol (dashed lines), respectively. Much more dramatic spectral changes are observed (solid *vs.* dashed) when compared with the isolated nanohole. **c** shows the corresponding normalized fluorescence spectra intensity ratio. The arrows indicate the wavelength shifts of corresponding constructive interference peaks determined by a small change in the refractive index when going from water to isopropanol ($\Delta n \approx 0.05$).

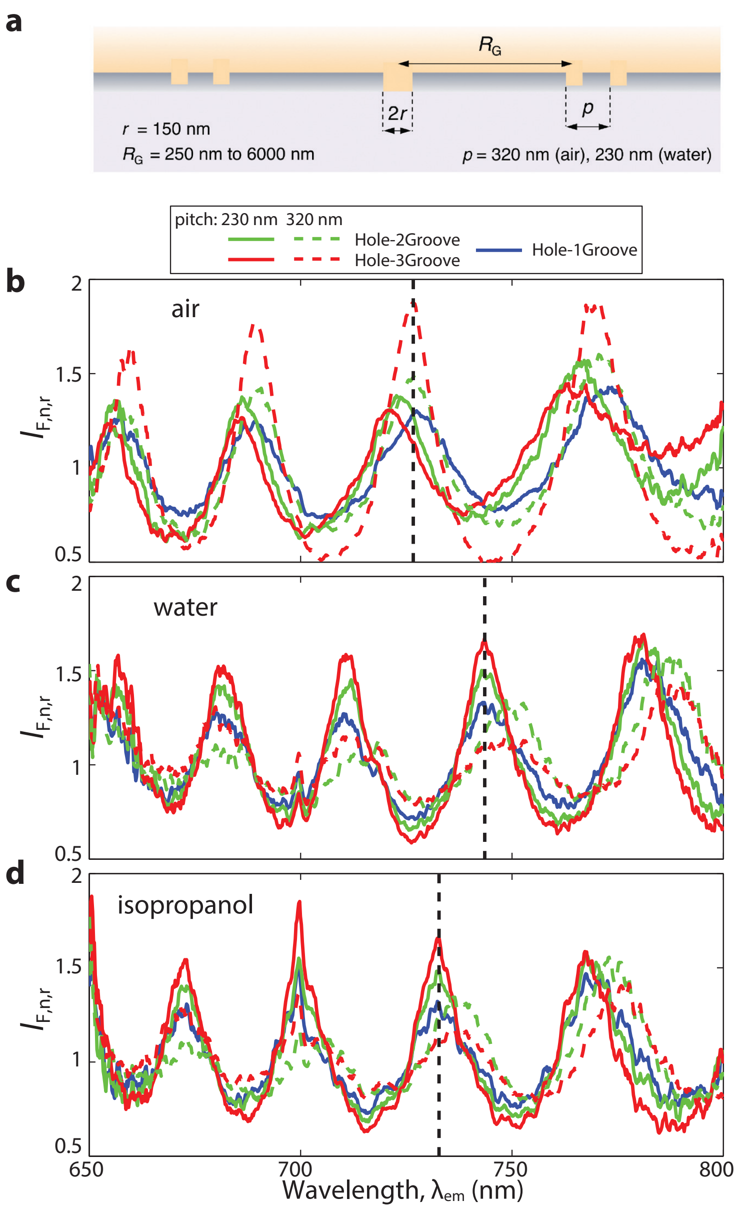

FIG. S11: **Comparison between biochemical sensing experiments for different values of groove number and grating pitch. a**, For the circular H-NG plasmonic interferometers, two different pitch values (i.e., $p = 320$ nm and 230 nm) were chosen by design. The value of $p = 320$ nm was selected to keep the SPPs reflected by different grooves in phase when the dielectric above the metal is air. In contrast, due to the change in the SPP complex refractive index occurring when flowing liquids on top of the metal surface (e.g. water, or isopropanol, which have similar refractive index values), a value of $p = 230$ nm will keep the reflected SPPs in phase at the nanoaperture location. **b-d** show the normalized fluorescence intensity ratio when the dielectric near the metal surface is air (**b**), water (**c**) and isopropanol (**d**) (for H-NG plasmonic interferometers with $R_G =$ 4.05 μm and varying N: blue $N = 1$, green $N = 2$, and red $N = 3$). When the dielectric layer on top of the interferometers is air (**b**) the resonant peak positions of H-2G and H-3G structures with grating pitch $p = 320$ nm (green and red dashed lines) match well the H-1G results (blue solid line), whereas in the presence of water (**c**) and isopropanol (**d**) the maxima observed when using a grating pitch of p =230 nm (green and red solid lines) match better the H-1G result, as denoted by the black dashed lines. This confirms that proper design of the pitch value is required for different dielectric materials. The wavelength shift observed in the peak positions in Fig. S4c compared with Fig. S11b for the 320nm pitch is due to a combination of a slightly different Cr^{3+}:MgO thickness and the addition of a 10nm-thick Al_2O_3 layer, which determine a slight yet observable change in the effective SPP refractive index.

X Fluorescence modulation of CdSe quantum dots embedded in plasmonic interferometers

A ∼20nm-thick layer of CdSe quantum dots was spin-coated onto the surface of slit-groove (Fig. S12a) and groove-slit-groove (Fig. S12b) plasmonic interferometers fabricated on a 300nm-thick gold film. Figures S12c,d show a clear (although weak) modulation of the fluorescence intensity transmitted through the slit of slit-groove and groove-slit-groove plasmonic interferometers as the result of SPP-mediated interference. The weak spectral modification can be explained as follows: (1) in the wavelength range of interest, the ohmic losses of SPPs on a gold surface are more significant than in silver; (2) the fluorescence spectral range is relatively narrow (FWHM∼30 nm compared with 600 nm emission wavelength). Nevertheless, these results show that the observed fluorescence spectra modification is not limited to Cr^{3+}:MgO emitters. It is however relevant to note that this latter system allows for plasmonic interferometry over a much wider spectral range and it is therefore more suitable to provide spectroscopic capabilities.

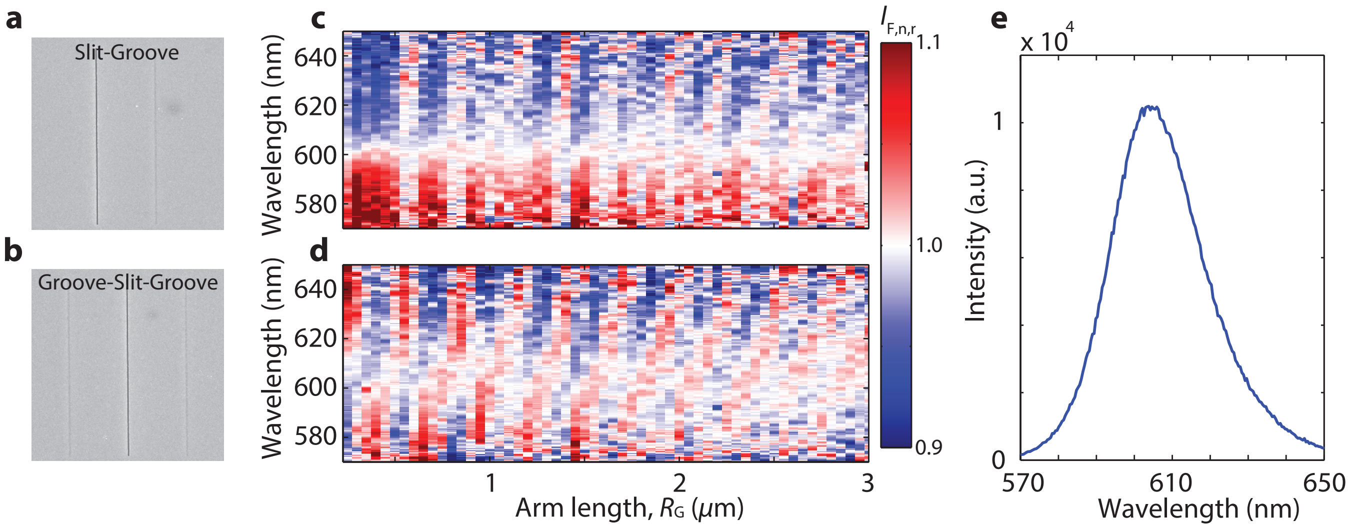

FIG. S12: **Color maps of normalized fluorescence intensity transmitted through the nanoslit of slit-groove and groove-slit-groove plasmonic interferometers coated with a ∼20nm-thick layer of CdSe quantum dots. a,b,** Representative SEM images of slit-groove (**a**) and groove-slit-groove (**b**) plasmonic interferometers. **c,d,** Color maps of normalized fluorescence intensity ratio in slit-groove (**c**) and groove-slit-groove (**d**) plasmonic interferometers as a function of emission wavelength and arm length. **e**, Representative transmitted fluorescence spectrum of CdSe quantum dots measured through an isolated single slit. The full width at half maximum is ∼30 nm.

[1] P. Lüsse, P. Stuwe, J. Schüle, and H.-G. Unger, J. Lightwave Technology **12**, 487 (1994).
[2] A. D. Rakic, A. B. Djurišic, J. M. Elazar, and M. L. Majewski, Appl. Opt. **37**, 5271 (1998).
[3] S. Karaveli, S. Wang, G. Xiao, and R. Zia, ACS Nano **7**, 7165 (2013).
